# Al6061 surface roughness and optical reflectance when machined by single point diamond turning at a low feed rate

**DOI:** 10.1371/journal.pone.0195083

**Published:** 2018-04-02

**Authors:** L. H. Li, N. H. Yu, C. Y. Chan, W. B. Lee

**Affiliations:** 1 The State Key Laboratory of Ultraprecision Machining Technology, Department of Industrial and Systems Engineering, The Hong Kong Polytechnic University, Hong Kong, China; 2 Shenzhen Branch of State Key Laboratory of Ultra-precision Machining Technology, The Hong Kong Polytechnic University Shenzhen Research Institute, Shenzhen, China; Massachusetts Institute of Technology, UNITED STATES

## Abstract

Ultra-precision face turning of Al6061 mirrors using single point diamond turning (SPDT) was undertaken to investigate the correlation between the surface roughness and reflectance. By reducing the feed rate, the optimal feed rate when the chip formation became unstable was studied. Most importantly, the impact on the roughness and reflectance was examined when the chip formation ceased to be continuous. It was determined that for a feed rate below 3 mm/min, the surface roughness always improved as the feed rate decreased, at the cost of a reduction of the normalized reflectance. The reduction of reflectance was determined to be the result of the lower material removal rates that led to a discontinuous chip formation.

## 1. Introduction

The Al6061 alloy is commonly used in ultra-precision machining because of its good machinability [1–3], and this aluminium is the preferred material for mirrors in spaceborne applications [4]. Such aluminium mirrors machined by ultra-precision machining technology have more stringent requirements on both the form accuracy and the surface roughness compared to that of optics lighting [5].

Previous studies have investigated the reflectance of metal alloy surfaces for different purposes, such as spaceborne applications or non-contact measurements. In these studies, factors such as the wavelength of the incident beam, the surface roughness and the material properties may affect the surface reflectance. Bennett and Porteus (1961) [6] studied the reflectance of an optically polished surface, at normal incidence, with surface roughness. They determined that the reflectance was wavelength-dependent. Whitley et al. (1987) [7] reported that as the roughness decreased, the specular reflectance increased for both nickel and stainless steel. Peiponen and Tsuboi (1990) further investigated the optical reflectance of nickel, aluminium, copper and brass and reported that the reflectance is strongly affected by the roughness. [8]. Yonehara et al. (2004) studied the experimental relationships between surface roughness, glossiness and colour of chromatic coloured metals [9]. The surfaces of their specimens were polished using abrasive paper. They determined that (1) the glossiness of their samples increased exponentially as the Ra value decreased; (2) the lightness value exhibited an inverse correlation between the Ra and specular reflectance (Gs (60°)); and (3) when Ra decreased, the surface colour became bluish as the reflectance of the long wavelength side decreased more significantly than that of the short wavelength side. Although different factors have been studied by researchers, there has been less focus on reflectance when the surface roughness machined by single point diamond turning (SPDT) is on the nanometre scale. Lei et al. (2010) determined that the reflectance decreased when the tool mark spacing was less than 6 μm in single point diamond machining (SPDM), but they did not study this phenomenon further [10]. Therefore, the reflectance characteristic of Al6061 mirrors when the surface roughness is in the nanometre range is investigated in this study, in order to optimize the machining parameters when turning the Al6061 mirror.

Single point diamond turning is a very well-known method for producing ultra-precise Al6061 mirrors with a roughness less than 10 nm and form errors less than 200 nm [11]. The surface roughness value of an ultra-precision face turned surface can be estimated by [12]:

$$Ra=\frac{0.032f^{2}}{RV^{2}}$$ (1)

where Ra is the ideal arithmetic surface roughness, f is the feed rate, R is the tool radius and V is the spindle speed. Ultra-precision face turning, even when the feed rate is very low, is in general agreement with Eq 1. Thus, a better surface finish can be expected with a decreasing feed rate. However, the same does not apply to the reflectance. When the feed rate is very low, decreasing the feed rate produces a decrease in the surface roughness; however, in this research, it was observed that the decrease in the roughness did not lead to a decrease in the reflectance of Al6061 mirrors when machined by SPDT.

Considering the function of mirrors, reflectance is always more important than surface roughness. Under such circumstances, surface roughness is simply used by convention to assist in indirectly gauging the reflectivity of an aluminium mirror. In general, good surface roughness of an aluminium mirror was assumed to be a guarantee of good reflectance. However, this assumption might fail when the feed rate is decreased to a certain level, such that continuous chip formation cannot be maintained. It is the prime objective of this research to study this phenomenon experimentally and systematically.

## 2. Materials and methods

### 2.1 Materials and machine

The Al6061-T6511 alloy used in this study was supplied by Kaiser Aluminum Ltd., USA. The turning was performed on an ultra-precision machine 450UPL (Moore Nanotech). The diamond tool used was C0.30mLGC from Contour Fine Tooling Ltd. The details of the materials used in the experiment and the turning parameters are shown in Table 1.

**Table 1:** Workpiece properties and cutting conditions.

Workpiece (Fig 1)	Al6061-T6511
Workpiece dimension (mm)	Diameter = 23 mm, Height = 15 mm
Number of samples	15
*Cutting conditions*	
Spindle speed (r/min)	1000
Feed rate (mm/min)	1,2,3,4,5,6,7,8,9,10,11,12,13,14,15
Depth of cut (μm)	5
Diamond tool tip radius (mm)	0.3128 (Fig 2)

**Fig 1:**
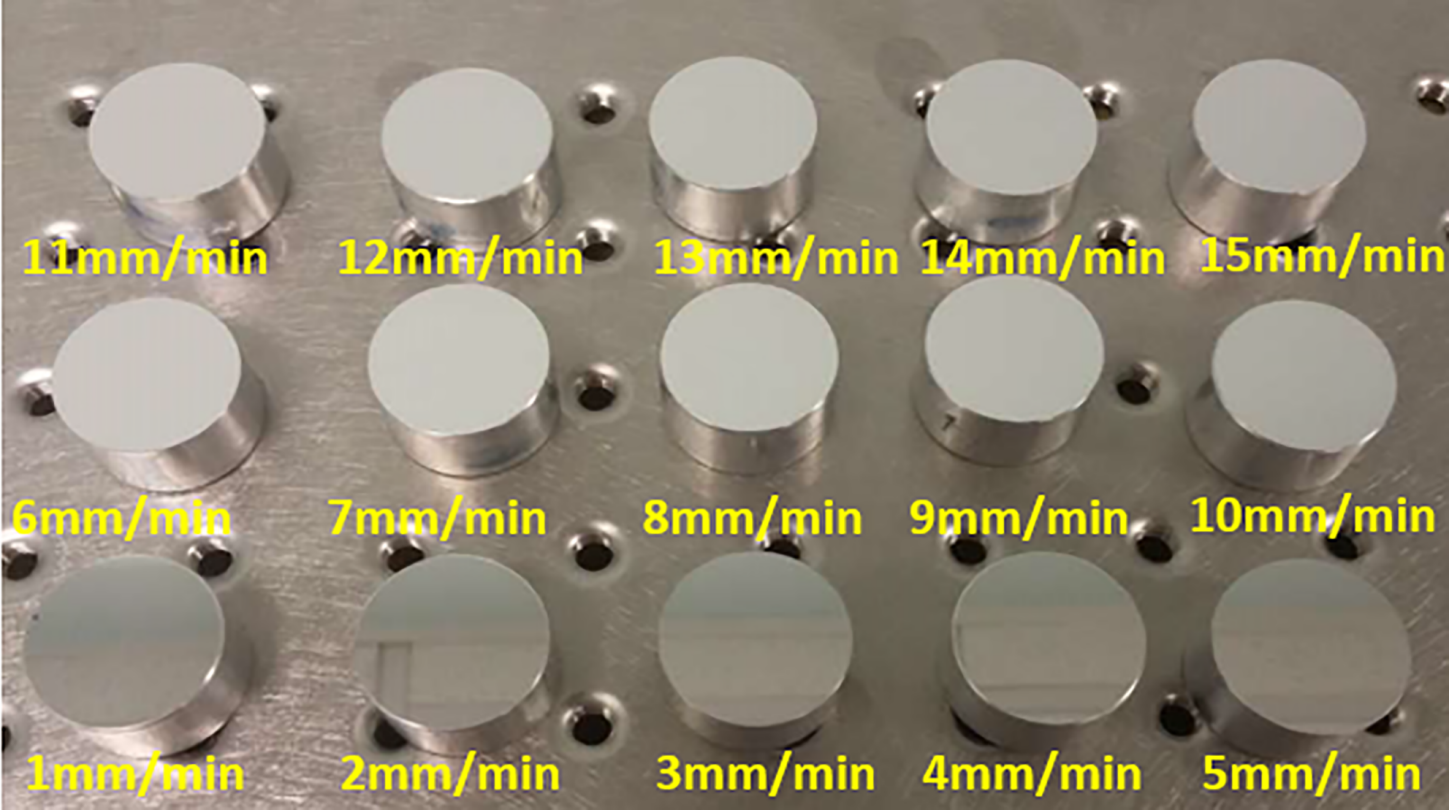
15 samples machined by SPDT at different feed rates.

**Fig 2:**
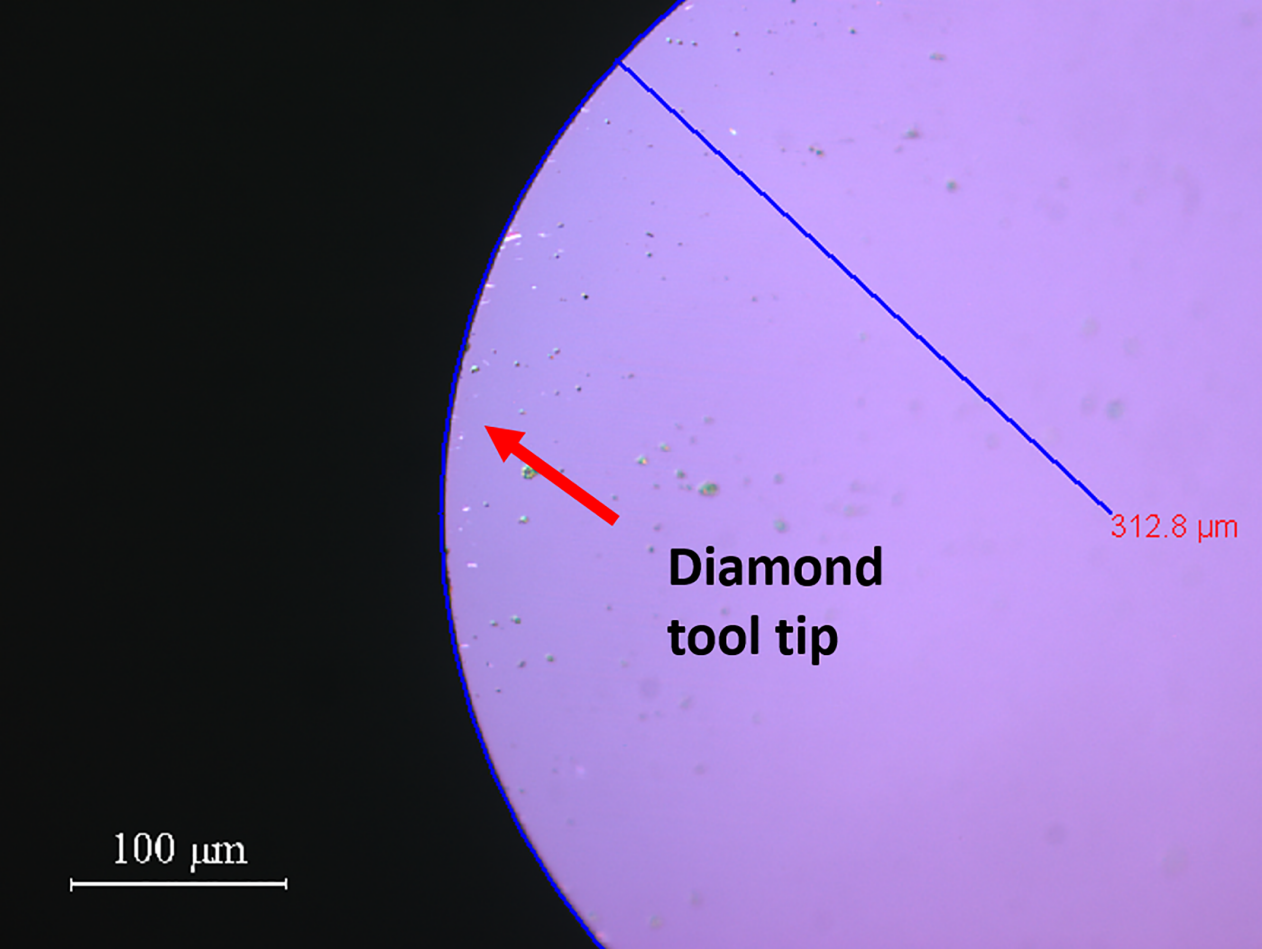
Measurement image of the diamond tool tip radius an Olympus BX60 microscope.

### 2.2 Characterisation methods

**Fig 3:**
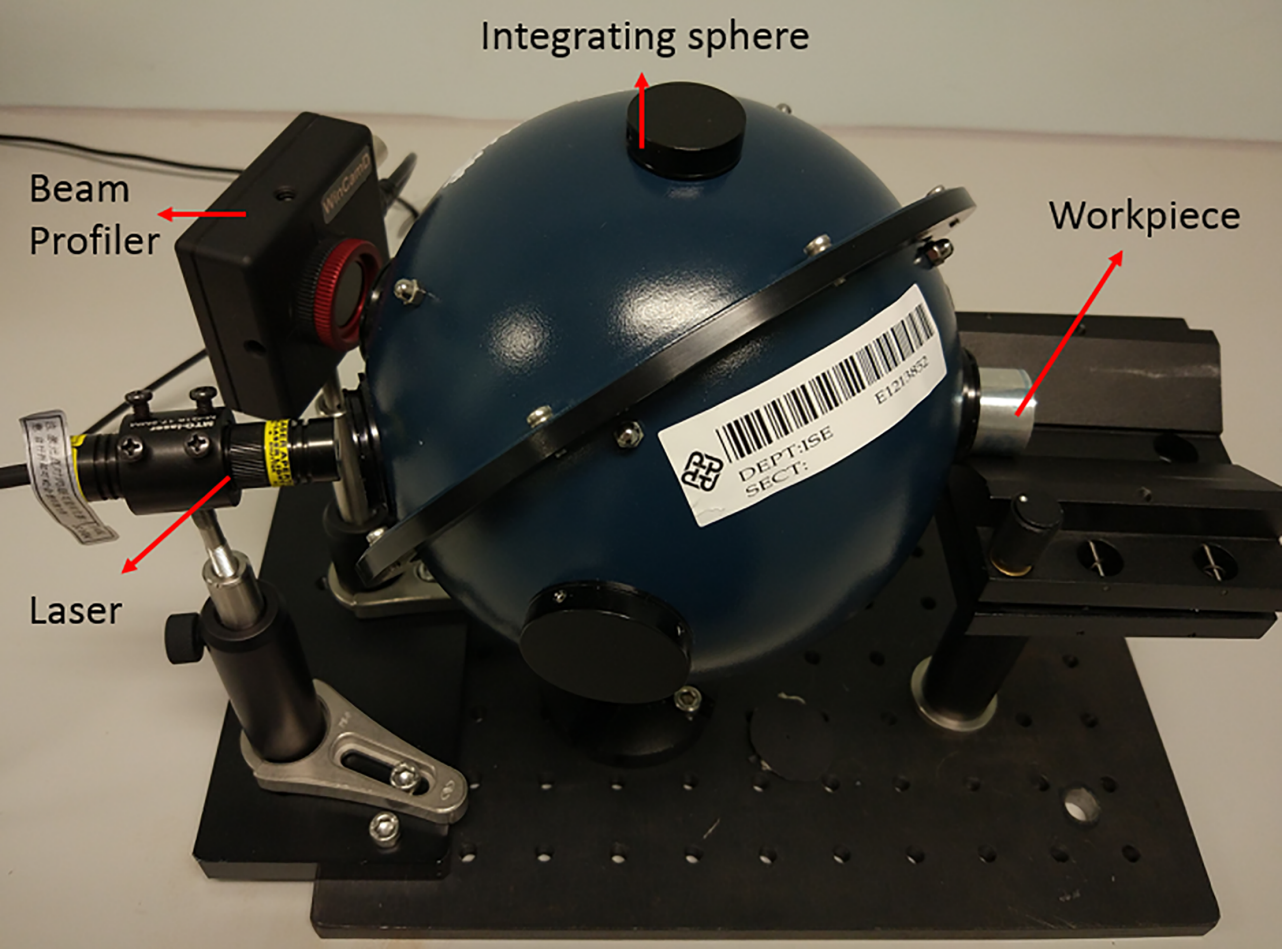
Reflectance measuring device.

First, the surface roughness was measured by a white light interferometer (Zygo Nexview) in the range of 700 ×700 μm^2^. Next, the reflectance of the same area on the samples was measured by the reflectance measuring system shown in Fig 3, in which a 100 mW laser was used on the workpiece surface (648 nm diode laser) and collected by a beam profiler (WinCamD UCD15). An integrating sphere was used to realize the photometric integration [13–14]. The reflectance was calculated by a comparison between the relative power I_o_ of the sample and a flat mirror I_r_ (protected silver mirror PF-10-03-P01 from Thorlabs, Inc). The relative reflectance can be expressed as R_r_ = I_o_/I_r_.

## 3. Results and discussion

**Fig 4:**
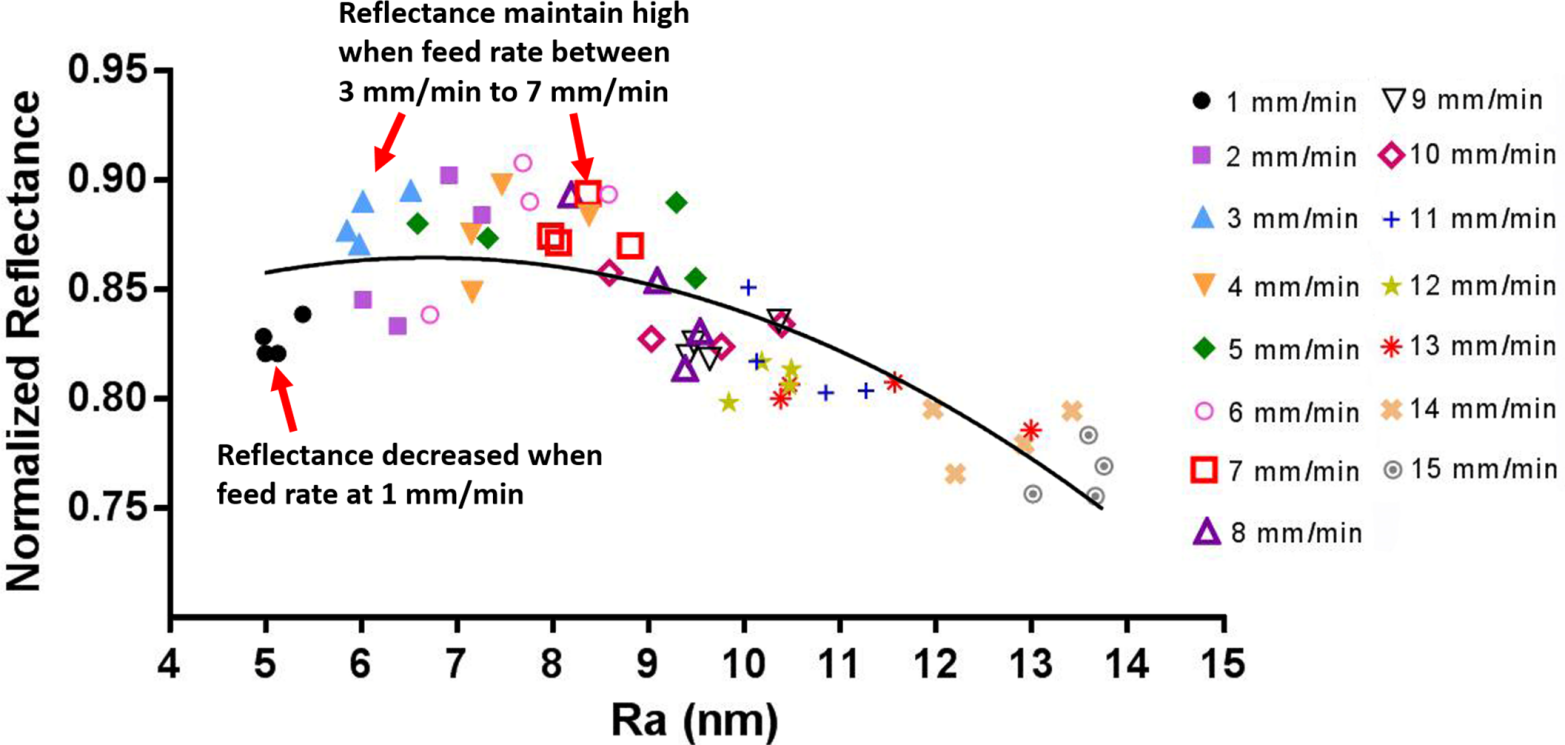
Results of normalized reflectance of 15 samples machined by single point diamond turning.

From the results, it was noted that when the feed rate was below 7 mm/min, the values of the normalized reflectance became unstable (Fig 4) and tended to fluctuate between 84% and 90%. It is possible that the chip formation could not be consistently maintained as continuous because the decrease in the feed rate may have caused a burnishing or rubbing dominated mechanism, instead of the cutting process [15].

**Fig 5:**
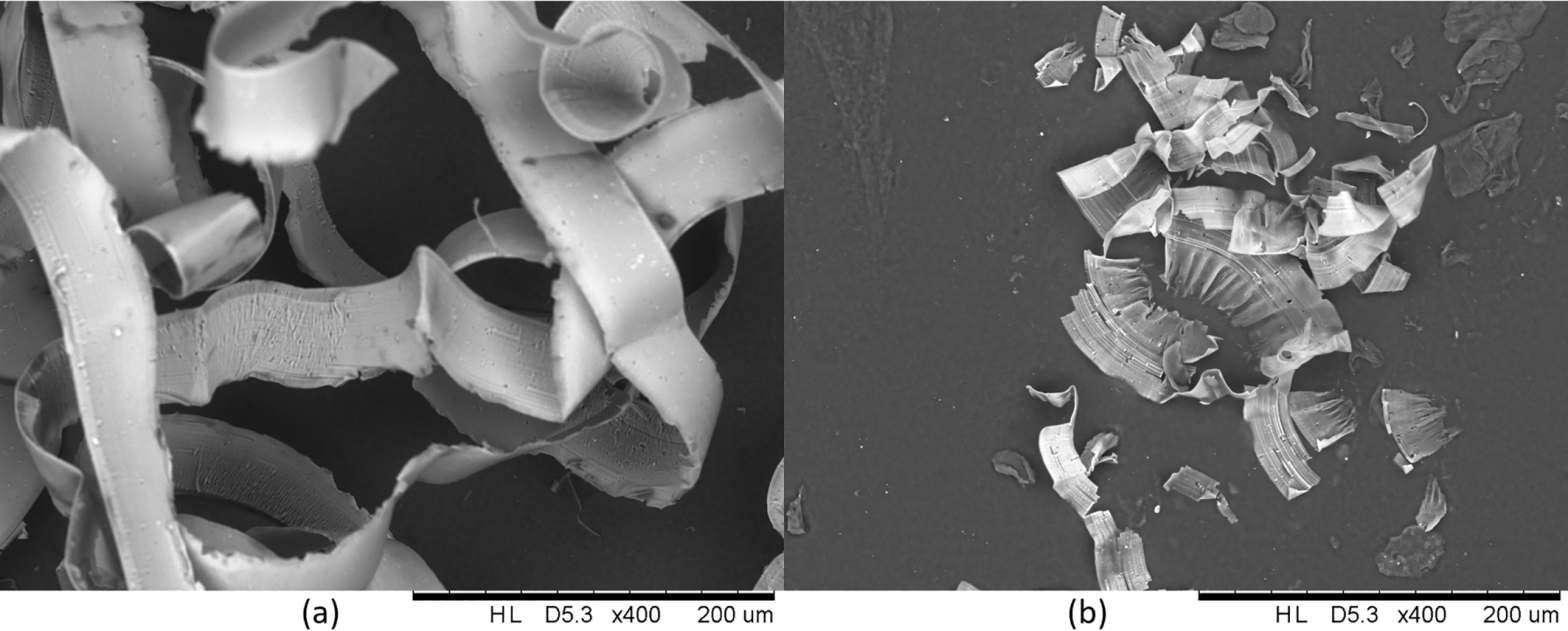
Chips measured by the Hitachi electron microscope TM3000, (a) feed rate is 7 mm/min, (b) feed rate is 3 mm/min.

The chip formation ceased to be continuous (Fig 5) when the feed rate was below 3 mm/min. Due to the chip formation becoming unstable, the decrease of the roughness did not result in a decrease in the normalized reflectance.

When the feed rate was further reduced from 3 mm/min to 1 mm/min, the surface roughness was reduced from 7 nm to 5 nm. However, such an improvement of the roughness is unfavourable on aluminium mirrors because it is achieved at the cost of a reduction in the normalized reflectance, which decreased from 90% to 82%.

**Fig 6:**
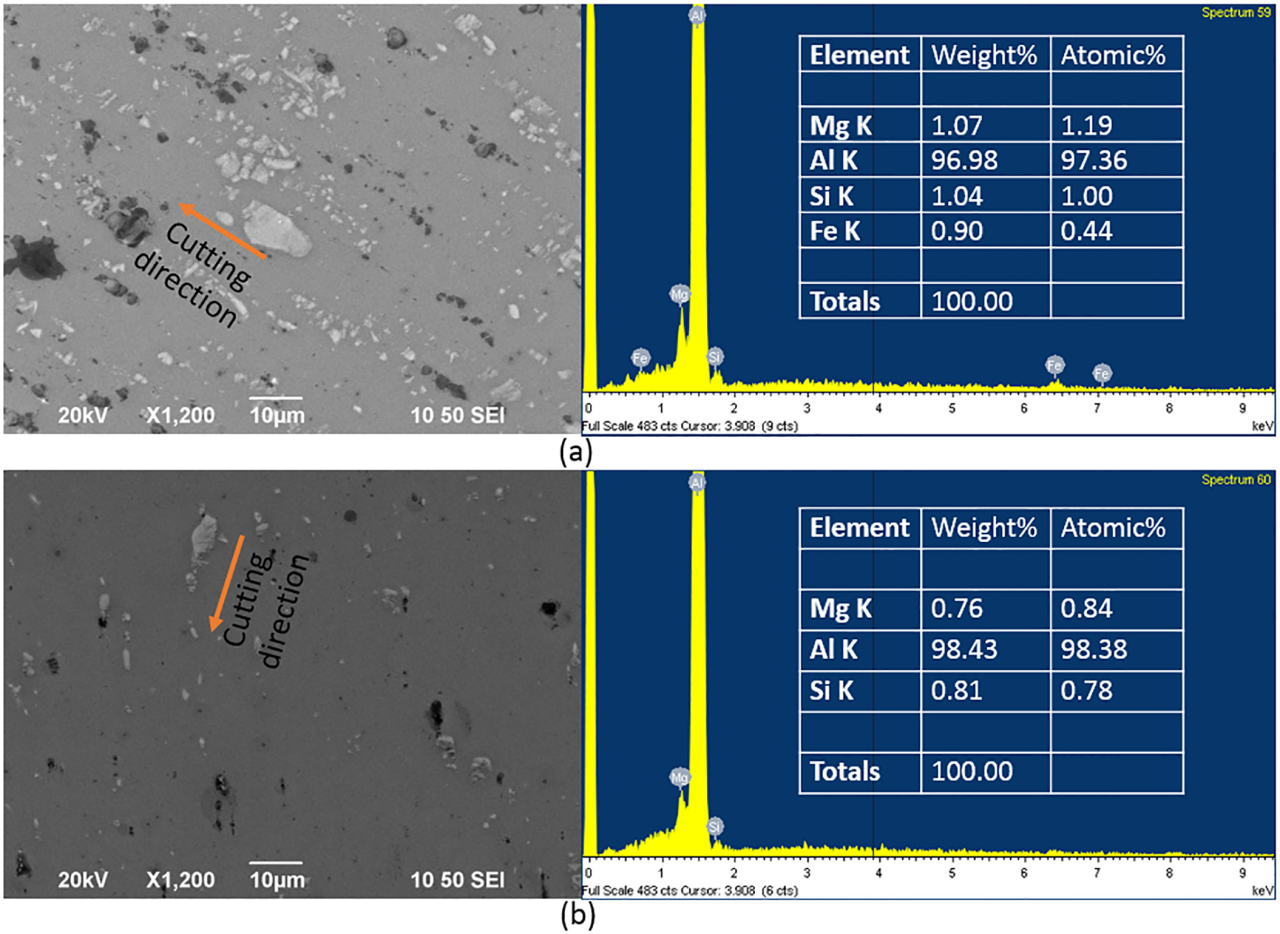
SEM images and EDX results of surfaces when the feed rates are 1 mm/min (a) and 7 mm/min (b), separately.

**Fig 7:**
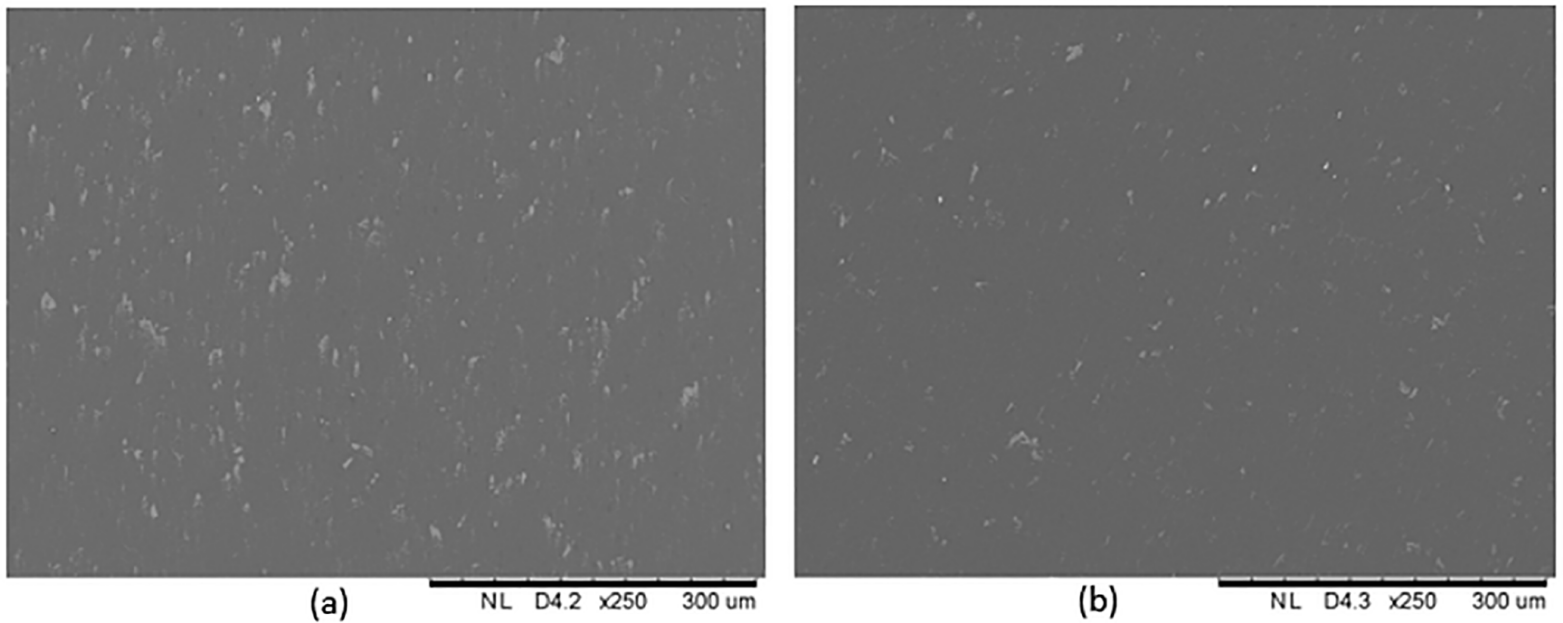
SEM images of surfaces when the feed rates are 1 mm/min (a) and 7 mm/min (b), respectively.

Thus, based on the above results, the optimal feed rate for face turning Al6061 alloy is between 3 mm/min and 7 mm/min for a depth of cut of 5 μm. Using EDX analysis (Fig 6), it was determined that the Mg_2_Si particles on the machined surface increased for samples cut at a lower feed rate, and the Mg_2_Si particles tended to align with the cutting direction when the surface was machined at a feed rate of 1 mm/min (see the arrow in Fig 6). For the sample cut at a feed rate of 1 mm/min, the Mg and Si content was 1.07 wt% and 1.04 wt%, respectively, and for the 7 mm/min sample, they were 0.76 wt% and 0.81 wt%, respectively. To exclude the effect of the used material itself, a verification cutting experiment was conducted by separately machining two new samples with a feed rate of 1 mm/min and 7 mm/min, in order to confirm that the amount of Mg_2_Si did increase when the feed rate was low. The results in Fig 7 indicate that the surface machined at a feed rate of 1 mm/min has more Mg_2_Si (white colour particles on the surface) than the surface machined at a feed rate of 7 mm/min; the reflectances were 0.84 (1 mm/min) and 0.93 (7 mm/min). This result confirmed that the reflectance of Al6061 alloy does not always monotonically decrease with the surface roughness.

**Fig 8:**
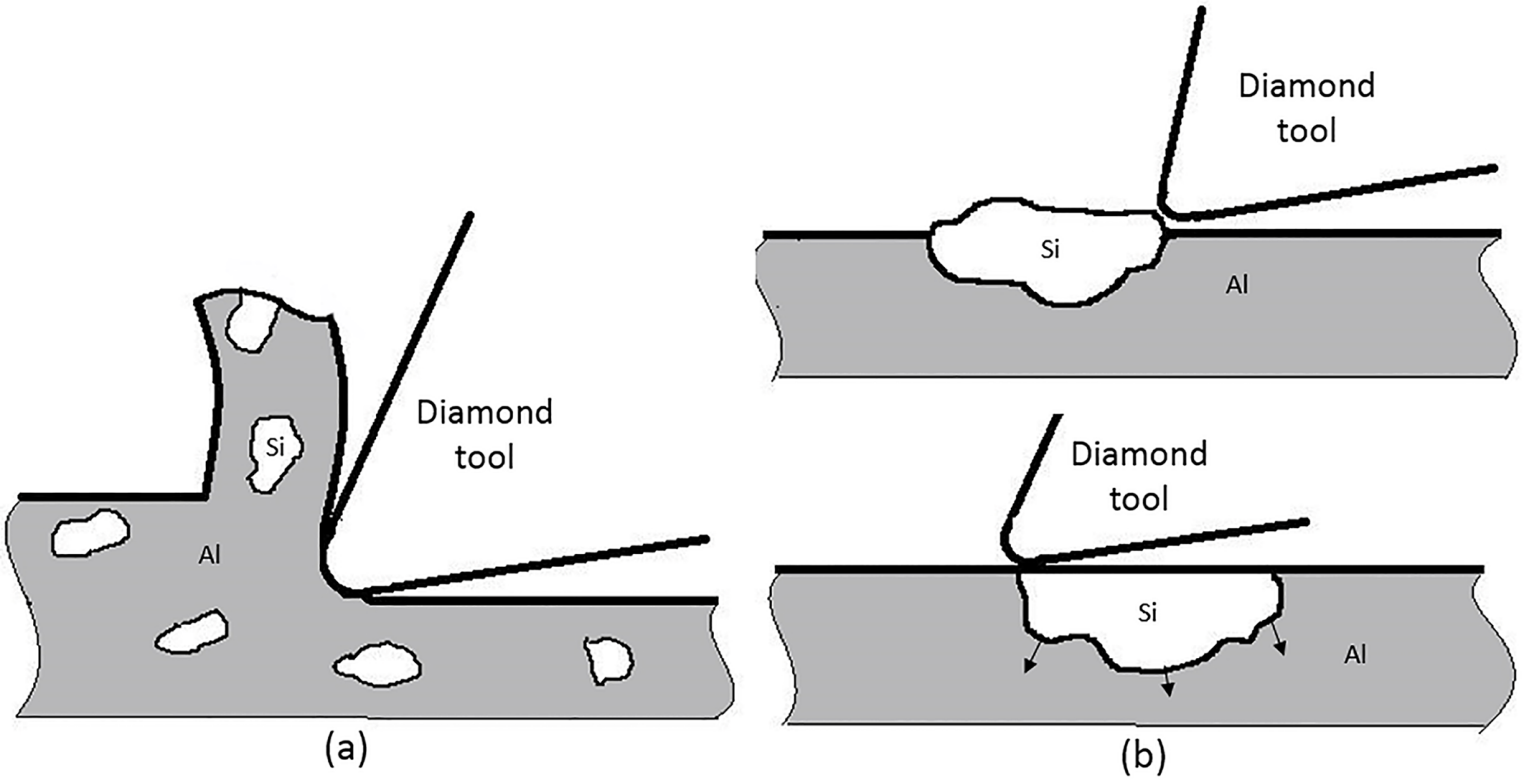
(a) Si phase is removed when feed rate occurs under optimal conditions (b) Si phase is embedded into surface when the feed rate is too low.

Since the SPDM temperature is approximately 30°C to 70°C when cutting metals with hardness (HRB) less than 90 [16–18], it is unlikely to cause any phase transformation on the surface because the cutting temperature is not high enough. Therefore, the reason for the changes in the amount of Mg_2_Si particles is likely due to the low feed rate (1 mm/min) causing a burnishing effect [15]: the Mg_2_Si particles were firmly pressed to the surface rather than being removed or knocked-out as shown in Fig 8, and these remaining Mg_2_Si particles caused the reduction of the reflectance of the Al6061 alloy. Further work, however, is needed to understand more about the possible mechanisms regarding this observation.

## 4. Conclusions

An assessment method was developed using an integrating sphere lumens measurement system to assess the reflectivity of a single point diamond turned surface of Al6061 alloy. This assessment method exhibited sufficient precision for assessing the reflectance of a machined surface.

Based on this method, it was determined that the reflectance of Al6061 alloy does not always monotonically increase with a decrease in the surface roughness. An optimal feed rate range exists (3 mm/min—7 mm/min in this study), and when the feed rate is larger than 7 mm/min, the Al6061 surface roughness is decreased as the feed rate decreases; therefore, this smoother surface causes the reflectance to increase. When the feed rate is decreased from 7 mm/min to 3 mm/min, the surface roughness continues to decrease, but the reflectance stabilizes, remaining at the same level. Below 3 mm/min, the chip formation is unstable, and the reflectance of the machined surface is unfavourably reduced as the feed rate decreases.

## Supporting information

S1 Table Supplement data for Fig 4. (XLSX)
